# The Treatment of Diphtheria

**Published:** 1892-10-15

**Authors:** 


### PADDINGTON GREEN HOSPITAL FOR CHILDREN.

### The Treatment of Diphtheria.

The symptoms of diphtheria vary so much in different cases, that no absolute rules can be laid down which are applicable to every case. There is, however, a certain amount of routine treatment pursued at every hospital, and the following notes indicate the ordinary practice at Paddington Green Children's Hospital.

On admission, the patient is put into a bed which is surrounded on all sides with thin curtains, and is situated in a warm and sheltered part of the ward. Great stress is laid on the maintenance of absolute rest in the horizontal position, and with the view of securing this no pillows are allowed; the child is laid on his back in bed, and the shoulders are firmly fixed. This fixation is secured by means of rings of broad webbing, which encircle each shoulder, passing under the arm-pit, and are joined together in front of the chest by another piece of webbing. A broad band of webbing is then passed through the rings posteriorly across the back of the child, and firmly fastened to the bed at each side. The limbs and head are thus left quite free, but the patient is unable to sit up or move about from side to side. The recumbent position is maintained during the whole course of the disease, and for at least a fortnight after the cessation of all acute symptoms. A gentle vapour of steam is directed over the bed from the end opposite the patient's head, and the kettle employed is "ventilating," with the funnel open at both ends, so that fresh air is constantly drawn in with the steam. To the water employed some medicament is added, such as eucalyptus or creasote. The following mixture is very frequently employed: Creasote, ʒj.; powdered acacia, ʒij.; carbolic acid lotion (1-20) to ℥ij. This is added to the kettle and is renewed every two hours. The temperature inside the tent is maintained at a uniform level of 60 deg. F. Suspended from the roof of the tent is a tray containing all the medicines, dressings, and instruments required in the treatment. No handkerchiefs are allowed for discharges, but pieces of old linen are employed, which are burned immediately after use. For the throat treatment, sublimate solution (one part in two thousand) is applied by spray or brush every two or three hours. The spray treatment, as a rule, disturbs the child less than the brush, and is usually preferred for that reason, but, where easily tolerated, the brush treatment allows of a more thorough and exact application of the antiseptic, and often of the removal of membrane which would otherwise be swallowed. When symptoms of nasal diphtheria are present all crusts are first removed by syringing the nostrils with warm solution of carbonate of soda (ten grains to one ounce). This is done by means of a soft rubber syphon tube, and without much difficulty, the stream of fluid is made to pass in at one nostril and out at the other. When the nostrils have been cleared the sublimate solution is substituted for the soda, and the irrigation is repeated every two, three, or four hours. In both throat and nose treatment the applications are not made so frequently during the night, if the child's rest be disturbed by so doing. The formula for the sublimate solution is as follows: ℞, Hydrargyri perchloridi, 1 part; sodii chloridi, 20 parts; glycerini, 200 parts; aquam destillatam, ad 2,000 parts. It is the custom for the house surgeon to apply a stronger solution of the sublimate thoroughly to the throat and nose at his morning and evening visits, the strength being one in five hundred, while the application of the weaker solution is left to the nurse.

The medicines ordinarily used are the perchloride of iron and chlorate of potash, and, on the appearance of cardiac irregularity or debility, strychnia and digitalis. The diet consists of milk, milk puddings, eggs, beef-tea, and Valentine's meat juice, and food is administered frequently, every two hours to a child under twelve months, and to all during the acute stage. If there is difficulty in swallowing, food is introduced directly to the stomach by means of a tube passed along the nostril and down the œsophagus. In cases of persistent sickness and vomiting the milk is peptonised with Fairchild's powders, and is usually found to be retained in this form. Alcohol in the form of brandy is the chief stimulant, and is given freely, the amount depending on the condition of the heart and pulse. During the convalescent stage tonics are given —cod-liver oil and iron, or cod-liver oil with hypophosphites, port wine, and cream (one to two ounces daily). After the acute stage is over, the child is still kept lying down for a fortnight, and then, if there are no signs of diphtheritic paralysis, he is propped up in bed, and in about ten days will be allowed to get up.

When there are signs of laryngeal disease, as shown by a persistent stridor, early tracheotomy is considered advisable. Chloroform is always used in those cases. When the trachea has been opened, the inside of the larynx is well feathered with sublimate solution (one in five hundred), with the view of disinfecting or detaching the membrane present. If the membrane has already spread below the opening, the same treatment is applied to the part of the trachea affected. Parker's tracheotomy tubes are preferred, but if there is much œdema of the neck before or after operation, Durham's tubes are found useful from their greater length of stem. When the tube has been introduced the wound is covered with a piece of lint smeared with iodo vaseline, and having an opening in the centre for the tube, and then over the outside is laid a couple of folds of carbolic gauze. After the operation, the nurse in charge keeps the trachea clear of inspissated mucus, &c., by feathering with a warm solution of carbonate of soda (twenty grains to one ounce), and removes and washes the inner tube if necessary. The outer tube is usually left unchanged for at least twenty-four hours, and longer, if possible, so as to allow of the closing up of the parts around it. After this has occurred, the outer tube is removed and cleaned once daily. If any membrane is present in the trachea or the wound, more frequent removals are necessary so as to allow of a thorough application of sublimate solution (one in five hundred) to the part affected. The tracheotomy tube is removed as soon as possible after the disappearance of all membrane, and the cessation of acute symptoms. A pad of carbolic gauze is fastened round the neck, and the child is thus able at first to use both the natural and the artificial openings, but the rapid closure of the latter soon re-establishes normal breathing. In some cases, owing either to laryngeal paralysis or laryngeal adhesions, early removal of the tube is impossible; for the former condition patience is required, and for the latter it will probably be found necessary to open up the larynx, and remove the adhesions.

If any symptoms of diphtheritic paralysis appear, the horizontal position of the patient is maintained, and the absolute rest thus secured is regarded as one of the most important methods of warding off a fatal attack of cardiac or respiratory paralysis. In the more serious cases hypodermic injections of strychnia (one-hundredth of a grain every four or eight hours) are employed, and with this atropia is often combined (one two-hundredth of a grain). Brandy is given in full doses, and food is administered frequently and in as large quantities as possible, either by the mouth or by the stomach tube. When the paralytic symptoms have passed off the patient is ordered solution of strychnia (two minims), and citrate of iron and quinine (four grains) three times a day as a tonic, and is sent to the convalescent home for a month.

